# Vascular Events and Carotid Atherosclerosis: A 5-Year Prospective Cohort Study in Patients with Type 2 Diabetes and a Contemporary Cardiovascular Prevention Treatment

**DOI:** 10.1155/2019/9059761

**Published:** 2019-12-22

**Authors:** Julien Vouillarmet, Charlotte Marsot, Delphine Maucort-Boulch, Benjamin Riche, Marjorie Helfre, Claire Grange

**Affiliations:** ^1^Hospices Civils de Lyon, Department of Endocrinology, Diabetes and Nutrition, Centre Hospitalier Lyon-Sud, Pierre-Bénite, France; ^2^Department of Biostatistics, Hospices Civil de Lyon, Lyon, France; ^3^Université Lyon I, Villeurbanne, France; ^4^CNRS, UMR 5558, Laboratoire Biostatistiques Sante, Pierre-Bénite, France; ^5^Hospices Civils de Lyon, Department of Vascular Medicine, Centre Hospitalier Lyon-Sud, Pierre-Bénite, France

## Abstract

**Background and Aims:**

European recommendations on cardiovascular prevention suggest that carotid atherosclerosis assessment by duplex ultrasonography could help in some cases to better assess CV risk. We investigated whether the presence of carotid atherosclerosis determined by duplex ultrasonography is associated with cardiovascular events in patients with type 2 diabetes and could therefore help to reclassify cardiovascular risk.

**Methods:**

Among 624 consecutive patients with type 2 diabetes and carotid atherosclerosis assessment by duplex ultrasonography between January and December 2012, 583 (93%) were included and followed up prospectively. The primary endpoint was the occurrence of cardiovascular events. The rate of new cardiovascular events was compared between patients with (*n* = 104) and those without (*n* = 479) prior cardiovascular events.

**Results:**

A total of new 104 cardiovascular events occurred in 72 patients (12.5%) during a mean ± SD follow-up period of 5.1 ± 1.6 years. At baseline, for 202 patients (34.6%), carotid evaluation was normal; 381 (65.4%) had a carotid atherosclerosis lesion. The presence of carotid atherosclerosis at baseline was not significantly associated with an increased risk of new cardiovascular events in both groups. The rate of new cardiovascular events was more than twice as high in patients with prior cardiovascular event than those without.

**Conclusion:**

Systematic carotid atherosclerosis assessment by duplex ultrasonography in patients with type 2 diabetes and a contemporary cardiovascular prevention treatment does not offer additional information as to the risk of cardiovascular events. This trial is registered with ClinicalTrials.gov (ID: NCT02929355).

## 1. Introduction

Although there has been notable improvement in the management of patients with type 2 diabetes and the decrease of cardiovascular (CV) events over recent years [1], patients with diabetes are still considered at a high risk of vascular events by the latest international guidelines on CV prevention [2, 3]. In European guidelines, nearly all patients with type 2 diabetes must be considered at a high CV risk with an LDL-C target below 100 mg/dL. Moreover, a subgroup at a very high risk has been identified, and for these, the LDL-C goal is below 70 mg/dL. This subgroup of patients includes those with prior CV events but also those without prior CV events with an additional important CV risk factor [4]. Similarly, the American Diabetes Association (ADA) [3] recommends at least a systematic moderate dose of statins according to age and risk factors that in practice makes nearly all patients with diabetes eligible, and a high dose of statin for those at a higher risk, i.e., when 2 additional CV risk factors are present and/or prior CV events. Despite these recommendations, individualisation of the vascular risk remains difficult and CV risk scores, based on traditional CV risk factors, do not predict well the presence of high CV risk and are not applicable to populations outside the development sample [5, 6]. Other tools exist to evaluate CV risk, such as the coronary artery calcium (CAC) score that is based on radiological imaging; it has a very high negative predictive value and seems interesting to avoid unnecessary treatment by statins, but its generalisation raises concerns about radiation exposure [7–9]. Another approach is to evaluate carotid atherosclerosis; for this, intima-media thickness of the carotid artery has been employed but is no longer recommended owing to its high variability and low intraindividual reproducibility [2]. Carotid plaque assessment by duplex ultrasonography (DUS), however, seems interesting as the presence of these is a strong predictor for future ischemic stroke but has also been associated with an increased risk of CV disease [10–19] and seems to better assess CV risk than the CV risk scores [20–23]. DUS is adapted to the evaluation of carotid plaques owing to the reproducibility of the measure and the safety of the procedure [24]. This has led to the suggestion in the most recent European recommendations that carotid atherosclerosis assessment by DUS could help in some cases to better assess CV risk [2]. Indeed, based on these published data [7–23], one can speculate that patients without prior CV events but with carotid atherosclerosis should be classified as at a very high risk as those with prior CV events. However, all but one [17] study with clinical follow-up [10–16, 18, 19] concerned cohorts without a contemporary CV preventive treatment—notably with a low use of statins, and a low prevalence of diabetes. The only study to have included patients who were well covered by statins and antiplatelet agents found that the presence of carotid atherosclerosis was an independent predictive factor of CV events, yet no subgroup analysis was performed to investigate whether this association was also found for those with diabetes who represented nearly half of the total study population [17]. In this context, there is currently insufficient proof to determine whether a systematic screening of carotid atherosclerosis by DUS in asymptomatic patients with diabetes and a contemporary preventive CV treatment is effective. Therefore, in the present study, we prospectively assessed whether there was an association between the presence of carotid atherosclerosis detected by DUS and new vascular events in patients with diabetes.

## 2. Material and Methods

### 2.1. Patients

All consecutive patients with type 2 diabetes who underwent DUS investigation of their extracranial carotid arteries from 1 January 2012 until 31 December 2012 in the Lyon teaching hospitals (Hospices Civils de Lyon, Lyon, France) were prospectively enrolled. Patients were referred to the endocrinology department for a systematic assessment of diabetes including identification of chronic complications. Patients with diabetes who were initially neurologically asymptomatic with respect to carotid artery disease were included.

At baseline, all patients had a complete workup (in the endocrinology department) concerning personal and familial history of vascular events, type of diabetes, duration of diabetes, glycaemic control, microangiopathy, and any medication including antihypertensive drugs, antidiabetic drugs, lipid-lowering drugs, and antiplatelet agents. Other CV risk factors were noted: fasting lipid profile, resting blood pressure, and smoking habit. Current smoking was defined as active smoking during the past 3 years. High blood pressure was defined as ≥140/85 mmHg [25]. Nephropathy was defined by the presence of at least one of the following signs: estimated glomerular filtration rate below 60 mL/min/1.73m^2^, positive microalbuminuria, or positive proteinuria.

Very high risk CV patients with a LDL-C target below 70 mg/dL according to European guidelines include those with prior CV events and those with an additional important CV risk factor (smoking, proteinuria, renal insufficiency, uncontrolled blood pressure above 180/110 mmHg, marked hypercholesterolaemia above 8 mmol/L (309 mg/dL)). Others were considered at high risk with a LDL-C target below 100 mg/dL [2].

According to ADA, patients with diabetes with an estimated high risk of CV event above 10% at 10 years include men or women aged ≥50 years who have at least one additional major risk factor (family history of premature atherosclerotic cardiovascular disease, hypertension, smoking, dyslipidaemia, or albuminuria) [3].

### 2.2. Carotid Atherosclerosis Assessment

**Figure 1:**
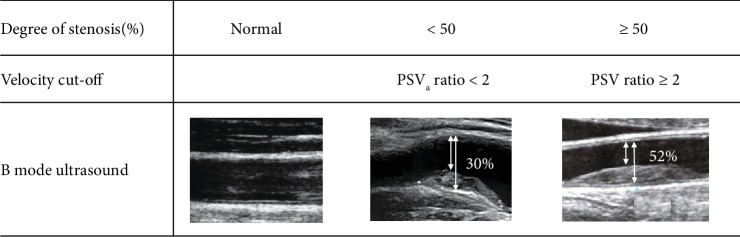
Duplex ultrasonography criteria. PSV: peak systolic velocity.

All included patients underwent assessment of carotid atherosclerosis by DUS. Both carotid arteries were examined using a B mode ultrasound (Siemens Acuson Antares, United Medical Instrument, San Jose, CA, USA) equipped with a 7-10 MHz high-resolution probe and operated by a trained technician. Peak systolic velocities were measured in the common carotid artery (CCA), carotid bulb, and internal carotid artery (ICA). A diagnosis of stenosis was made when velocity criteria consistent with the existence of plaques and the narrowing of the lumen were observed. The velocity cut-off described below permits to estimate the degree of stenosis with a good correlation to North American Symptomatic Carotid Endarterectomy (NASCET) criteria [26]. The most severe lesion for each patient was selected, and the thickness of these were classified as recommended as normal, plaque thickness < 50% of the diameter without acceleration (ICA/CCA peak systolic velocity ratio < 2), and thickness ≥ 50% with acceleration (ICA/CCA peak systolic velocity ratio ≥ 2) [27] (Figure 1).

New CV events were prospectively assessed: CV-related death, acute myocardial infarction, stroke or transient ischemic attack, coronary revascularisation, carotid revascularisation, or lower limb revascularisation. Major adverse cardiac event (MACE) was defined as either CV-related death, acute myocardial infarction, or stroke. Patients were followed-up in routine care without systematic CV exams according to guidelines, but all new CV events were documented on medical report.

### 2.3. Regulatory Aspects

The investigations were carried out in accordance with the principles of the Declaration of Helsinki. The study was registered with the institutional office of the national data protection agency (Commission Nationale de l'Informatique et des Libertés; ID: 15-021) and registered on ClinicalTrials.gov (ID: NCT02929355). All patients were provided with written information. In accordance with legislation in place at the time of the study in France, ethics approval was not required, and oral informed consent was collected from all participants.

### 2.4. Statistical Analysis

The primary outcome was the first occurrence of any of the following new CV events: CV-related death, acute myocardial infarction, stroke or transient ischemic attack, coronary revascularisation, carotid revascularisation, or lower limb revascularisation. Variables were described using the mean and standard deviation (SD) for continuous variables, counts and percentages for categorical ones. Univariate proportional hazards Cox models were first fitted with regard to CV events for the following variables: age, current smoking, antiplatelet treatment, blood pressure ≥ 140/85 mmHg, nephropathy, and the presence of carotid atherosclerosis at baseline for the 479 patients without prior CV events. Analyses were repeated for the 104 patients with prior CV events. Then, multivariate models were built including significant and/or clinically relevant variables. For each model, the proportional assumption was checked, and hazard ratios with corresponding 95% confidence interval were estimated. Nonlinearity effects of covariates (especially age) were assessed. A log-rank test was used to compare survival curves between all strata. Statistical significance was defined as a value of *p* < 0.05. Statistical analysis was performed using the SAS software, version 9.1.3 (SAS Institute, Cary, NC, USA).

## 3. Results

### 3.1. Characteristics of the Population

**Figure 2:**
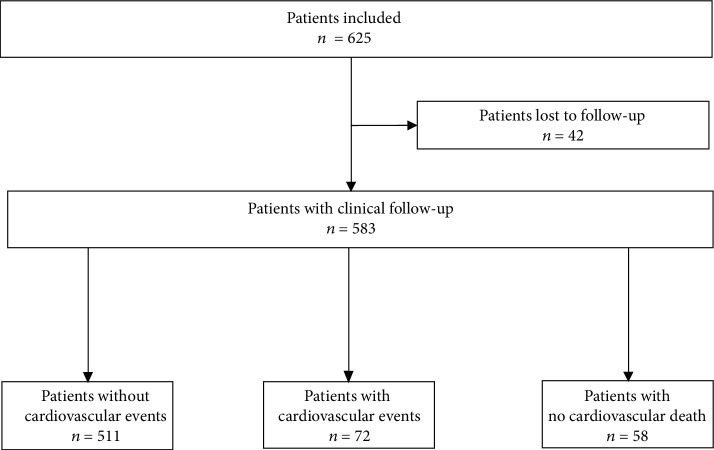
Study flow chart.

A total of 625 patients were included; 583 had clinical follow-up data available, and 42 patients were lost to clinical follow-up (Figure 2). Among those with clinical follow-up, there were 68 deaths. The mean ± SD duration of which was 5.1 ± 1.6 years, and the median (range) was 5.7 (0.2-7.6) years; there was no significant difference in baseline characteristics between patients with or without follow-up.

Baseline characteristics of the population are described in Table 1. A total of 104/583 patients (18%) had a prior CV event, and among those without a prior CV event, 313/479 patients (65%) had an estimated risk of CV event at 10 years greater than 10% according to the ADA [3] and 160/479 (33%) were considered at a very high CV risk according to European guidelines and therefore a LDL-C target below 70 md/dL [2]. Patients with prior CV events were significantly older than patients without prior CV events, had a longer duration of diabetes, had a more frequent use of insulin, and had more frequent microvascular complications. They were significantly more frequently covered by lipid- and blood pressure-lowering therapy; they most frequently had an antiplatelet agent, and LDL-C was significantly lower.

Indication of DUS at baseline was a systematic screening for 385 patients (66%) and a follow-up of known carotid atherosclerosis lesion for 198 patients (34%). At baseline, 202 patients (34.6%) presented a normal carotid DUS, 369 had a stenosis < 50% (63.2%), and 12 had a stenosis ≥ 50% (2.0%); 1 patient had a stenosis > 70%.

### 3.2. New Cardiovascular Events

**Figure 3:**
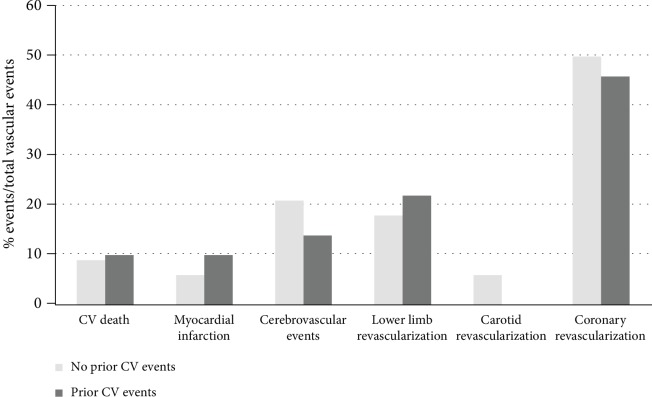
Distribution of new vascular events in patients with or without prior cardiovascular (CV) events.

During follow-up, 104 CV events occurred in 72 patients (12.5% of the 583 patients with follow-up; 3.5%/patient/year); 70 CV events in those without prior CV events (2.8%/patient/year) and 34 in those with prior CV events (6.2%/patient/year). Among these events, 33 MACE occurred in 32 patients (1.0%/patient/year); 24 MACE occurred in those 23 patients without prior CV event (0.9%/patient/year) and 9 in those with prior CV events (1.7%/patient/year). There were also 68 revascularisations (coronary, *n* = 49; lower limb, *n* = 21; or carotid, *n* = 1). Over half of coronary revascularisations were performed after cardiac symptoms (chest pain at rest or exercise, angina, acute pulmonary oedema, or dyspnea; 29/49, 59%). A minority was performed after myocardial infarction (7/49, 14%) and a quarter after silent myocardial ischemia screening (13/49, 27%). Half of the lower limb revascularizations were performed after diabetic foot syndrome (12/21, 57%), while the others were performed because of intermittent claudication (8/21, 38%) or after systematic screening (1/21, 5%). Only one carotid revascularisation was performed during the follow-up after a transient ischemic attack. Only 2 patients with prior CV events presented a ST elevation myocardial infarction (STEMI) during follow-up and none of them a non-STEMI (NSTEMI). Distribution of vascular events in patients with or without prior CV events is reported in Figure 3. There was no significant difference in the distribution of new CV events between the two groups. The mean ± SD interval between baseline carotid DUS and the first CV event was 3.02 ± 1.98 years.

**Figure 4:**
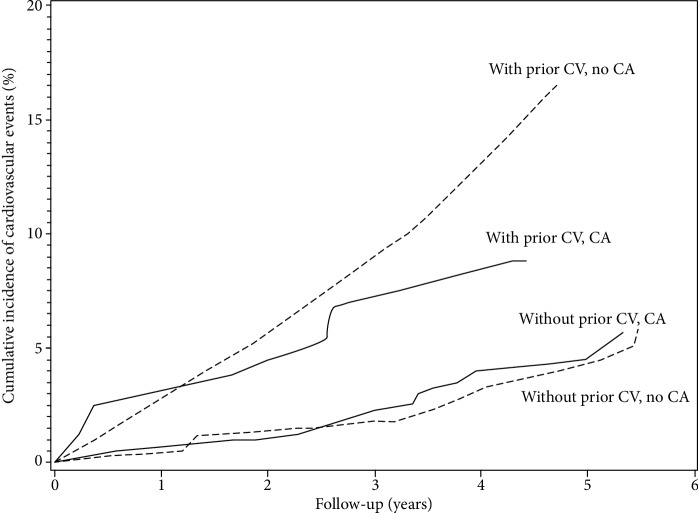
Cumulative incidence of new cardiovascular events. CV: cardiovascular event; CA: carotid atherosclerosis. (log-rank test to compare survival curves between all strata, *p* = 0.189).

Rate of new CV events in patients with or without prior CV events according to the presence or absence of carotid atherosclerosis is reported in Figure 4. Among those patients with prior CV events, 34 CV events occurred in 21/104 patients, including 29 CV events in 17/84 patients with carotid atherosclerosis and 5 CV events in 4/20 patients without carotid atherosclerosis. Concerning these 21 patients, 15 had nephropathy (71%), 4 smoked (19%), 19 had a lipid-lowering therapy (90%), 20 had a blood pressure-lowering therapy (95%), and 17 had an antiplatelet therapy (81%). LDL-C goal < 70 mg/dL was achieved in 6 patients (33%), and blood pressure was uncontrolled in 4 patients (13%); these 4 patients had blood pressure-lowering therapy.

Among those patients without prior CV events, 70 CV events occurred in 51/479 patients, including 48 CV events in 36/299 patients with carotid atherosclerosis and 22 CV events in 15/180 patients without carotid atherosclerosis. Concerning these 51 patients, 27 had nephropathy (53%) and 10 smoked (20%). Nearly half of patients had antiplatelet therapy (*n* = 25; 49%), 41 had a lipid-lowering therapy (80%), and 44 had a blood pressure-lowering therapy (86%). A total of 19 patients were at a very high CV risk according to European guidelines [3] with a LDL-C target < 70 mg/dL (no patient was at this target), and 14 were at a high CV risk with a LDL-C target < 100 mg/dL (4 patients were at this target, 29%).

In both univariate and multivariate analysis, only age was significantly associated with new CV events in both groups, i.e., patients with prior CV events and those without prior CV events (Tables 2 and 3).

## 4. Discussion

The present study found that there was a low rate of carotid atherosclerosis progression and no association between the presence of carotid atherosclerosis and CV events in patients with type 2 diabetes and a contemporary preventive CV treatment. Moreover, the rate of new CV events was threefold lower in patients without prior CV events, even when associated with carotid atherosclerosis, compared to those with prior CV events with or without carotid atherosclerosis.

As observed elsewhere among patients with diabetes [28, 29], a high prevalence of carotid atherosclerosis was found, but only a few patients had a severe lesion ≥ 50%. Contrary to other studies [10–19, 30], no association between the presence of carotid atherosclerosis and CV events was found herein among patients with diabetes and mostly at a high CV risk. To explain this discrepancy, it is important to note that most of the previous studies concerned patients with a low to intermediate CV risk with a low use of statin and only a small proportion of included patients had diabetes (from 0 to 21%) [10–16, 18, 19]. Furthermore, even when CV preventive treatment is described in these reports, there is no information on the number of patients who reach the target for LDL-C and blood pressure. For patients at a high CV risk with carotid atherosclerosis assessment (*n* = 23 364), the REACH study found a 22% increased risk of CV events associated with the presence of carotid lesion that after *a priori* stratification was found only among those with prior CV events [17]. The potential association with diabetes (41% of the population) with the presence of carotid atherosclerosis was not investigated. Finally, two small studies that included only patients with diabetes and a low to intermediate risk of CV events found that carotid plaque was less accurate than the CAC score to predict coronary disease assessed by coronary computed tomography angiography [31] and CV events [32]. Taken together, the exclusive inclusion of patients with diabetes and a high estimated CV risk receiving contemporary preventive treatments herein could explain the absence of association between carotid atherosclerosis and CV events observed in other studies.

Although a comparison between studies is difficult due to heterogeneity in the definition of CV events, the findings herein concerning the rate of CV events seem in line with that reported in the literature. Focusing on the rate of MACE (CV-related death, stroke, and myocardial infarction) in patients with type 2 diabetes without prior CV events, incidence of events in the present study was low (1%/patient/year) and close to that reported in the DIAD study (0.6%/patient/year) [33]. The rate of MACE herein is higher for patients with prior CV events (1.7%/patient/year), but lower than observed in literature (from 3.5% to 7.9%/year) [34]. Another point to consider is that patients with prior CV events have often more advanced disease than those without, as reported herein, which partly explains the higher risk of CV events in this population. Conversely, they had also a more aggressive CV preventive treatment and a lower LDL-C.

The present study does, however, has several limitations. The size of the cohort is relatively small which could affect interpretation; however, the rate of CV events for patients without prior CV event but carotid atherosclerosis was much lower than those with prior CV events and does not justify a substantial modification in terms of preventive CV treatment. The second point is that, although the majority of the patients included herein were at a high CV risk with widespread use of CV preventive treatment, as many patients were not on target as required by recent guidelines [2]. It is, however, important to note that this is an observational study without recommendation to adjust CV treatment. Studies that observe real-life practice found similar results; 43 to 58% of patients at a high CV risk and 72 to 78% of patients at a very high risk were still above LDL-C target despite the use of statins [35–37]. In the same way, it was observed that a quarter of patients with an indication for treatment were not receiving statins and that the intensity of statin was not sufficient in nearly half of cases [38] underlining the need to be more aggressive in terms of CV risk factor control. However, as not all patients had optimal CV treatment, the rate of CV events could be further reduced in the population studied herein. Another aspect to consider is that the cohort included a low rate of significant carotid stenosis as observed elsewhere [28, 29, 39, 40], and we cannot exclude a link between CV risk of carotid atherosclerosis for patients with stenosis > 50% or >70%.

## 5. Conclusions

To conclude, there was no significant association between the presence of carotid atherosclerosis and the occurrence of new CV events in patients with type 2 diabetes. Therefore, screening using carotid DUS may not be very informative for the assessment of CV risk in patients with type 2 diabetes who are at high CV risk based on usual risk factors.

## Figures and Tables

**Table 1:** Characteristics of the population at baseline.

	Total population (*n* = 583)	Patients without prior CV^a^ events (*n* = 479)	Patients with prior CV events (*n* = 104)	*p* value
Age (years)	63.5 ± 11.1	62.5 ± 11.2	67.8 ± 9.2	<0.01
Male sex	332 (57.6)	258 (54.7)	74 (72.2)	0.002
HbA1c (%)	8.5 ± 2.0	8.5 ± 2.0	8.9 ± 1.9	0.117
HbA1c (mmol/mol)	69 ± 19	69 ± 19	74 ± 18	
Diabetes duration (years)	14.0 ± 9.9	13.0 ± 9.5	18.8 ± 10.4	<0.001
BMI^b^ (kg/m^2^)	31.6 ± 6.6	31.7 ± 6.6	31.0 ± 6.8	0.262
Current smoker	124 (22.3)	108 (23.7)	16 (16)	0.086
Mean triglycerides (mg/dL)	174 ± 132	170 ± 117	193 ± 186	0.111
HDL-C^c^ (mg/dL)	44 ± 13	45 ± 13	41 ± 14	0.011
LDL-C^d^ (mg/dL)	102 ± 35	104 ± 35	90 ± 35	<0.001
Blood pressure ≥ 140/85	154 (26.8)	130 (27.6)	24 (23.1)	0.312
Lipid-lowering therapy	421 (73.1)	327 (69.3)	94 (90.4)	<0.001
Statin	394 (68.4)	303 (64.2)	91 (87.5)	<0.001
Blood pressure-lowering therapy	456 (79.2)	354 (75.0)	102 (98.1)	<0.001
ACEi^e^/ARB^f^	399 (69.3)	306 (64.8)	93 (89.4)	<0.001
Antiplatelet therapy	262 (46.1)	172 (37.1)	90 (86.5)	<0.001
Diabetes treatment				<0.001
Insulin	122 (21.3)	89 (19.0)	33 (32.0)	
OAD^g^/incretin	227 (39.7)	206 (43.9)	21 (20.4)	
OAD/incretin and insulin	205 (35.8)	158 (33.7)	47 (45.6)	
Lower extremity arterial disease	68 (11.8)	42 (8.9)	26 (25.0)	<0.001
Previous cardiovascular event	89 (15.5)	0 (0)	89 (85.6)	<0.001
Previous cerebrovascular event	25 (4.3)	0 (0)	25 (24.0)	<0.001
Retinopathy	176 (30.6)	131 (27.8)	45 (43.3)	0.003
Neuropathy	235 (40.8)	177 (37.5)	58 (55.8)	0.001
Nephropathy	227 (39.4)	173 (36.7)	54 (51.9)	0.005

Data are *n* (%) or mean ± SD. ^a^CV: cardiovascular; ^b^BMI: body mass index; ^c^HDL-C: high-density lipoprotein cholesterol; ^d^LDL-C: low-density lipoprotein cholesterol; ^e^ACEi: angiotensin-converting enzyme inhibitors; ^f^ARB: angiotensin II receptor blocker; ^g^OAD: oral antidiabetic drugs.

**Table 2:** Factors associated with new cardiovascular events in patients without prior cardiovascular events.

	Univariate model		Multivariate model	
	HR (95% CI)	*p* value	HR (95% CI)	*p* value
Carotid atherosclerosis	1.43 (0.78–2.63)	0.247	1.05 (0.50–2.53)	0.735
Age	1.77 (1.34–2.35)	<0.001	1.67 (1.21–2.29)	0.002
Current smokers	0.69 (0.32–1.47)	0.335	1.13 (0.55–2.53)	0.773
Nephropathy^a^	2.04 (1.16–3.58)	0.013	1.40 (0.77–2.53)	0.264
Antiplatelet treatment	1.73 (0.98–3.04)	0.058	1.38 (0.72–2.62)	0.331
Blood pressure ≥ 140/85 mmHg	2.35 (1.33–4.15)	0.003	1.60 (0.87–2.95)	0.130

CI: confidence interval; HR: hazard ratio. ^a^Defined by the presence of at least one of the following: eGFR < 60 mL/min/1.73m^2^, positive microalbuminuria, or positive proteinuria.

**Table 3:** Factors associated with new cardiovascular events in patients with prior cardiovascular events.

	Univariate model		Multivariate model	
	HR (95% CI)	*p* value	HR (95% CI)	*p* value
Carotid atherosclerosis	1.30 (0.44–3.86)	0.436	1.24 (0.35–5.93)	0.738
Age	2.37 (1.35–4.16)	0.003	2.07 (1.11–3.85)	0.022
Current smokers	1.46 (0.49–4.42)	0.498	1.89 (0.59–5.93)	0.283
Nephropathy^a^	2.57 (0.97–6.63)	0.051	1.27 (0.36–4.50)	0.714
Antiplatelet treatment	0.45 (0.15–1.37)	0.058	0.57 (0.15–2.13)	0.402
Blood pressure ≥ 140/85 mmHg	0.77 (0.26–2.30)	0.647	0.73 (0.24–2.24)	0.578

CI: confidence interval; HR: hazard ratio. ^a^Defined by the presence of at least one of the following: eGFR <60 mL/min/1.73m^2^, positive microalbuminuria, or positive proteinuria.

## Data Availability

The data used to support the findings of this study are available from the corresponding author on reasonable request.

## Abbreviations

CAS: Coronary calcium score
CCA: Common carotid artery
CV: Cardiovascular
DUS: Duplex ultrasonography
ICA: Internal carotid artery
IMT: Intima-media thickness
MACE: Major adverse cardiac events
NASCET: North American Symptomatic Carotid Endarterectomy.
